# Age-related effects on a novel dual-task Stroop paradigm

**DOI:** 10.1371/journal.pone.0247923

**Published:** 2021-03-02

**Authors:** Nathan Ward, Erika Hussey, Reem Alzahabi, John G. Gaspar, Arthur F. Kramer

**Affiliations:** 1 Department of Psychology, Tufts University, Medford, MA, United States of America; 2 Center for Applied Brain and Cognitive Sciences, Medford, MA, United States of America; 3 U.S. Army Combat Capabilities Development Command Soldier Center, Natick, MA, United States of America; 4 National Advanced Driving Simulator, University of Iowa, Iowa City, IA, United States of America; 5 Center for Cognitive & Brain Health, Northeastern University, Boston, MA, United States of America; 6 Beckman Institute, University of Illinois, Champaign, IL, United States of America; University of Padova, ITALY

## Abstract

The Stroop task is a traditional measure of cognitive control processes, yet results remain mixed when it comes to assessing age-related differences perhaps in part due to strategies participants use to reduce inhibitory control demands required for success on the task. Thirty-three older adults and 34 younger adults completed a Baseline (traditional, single-task) version of Stroop, followed by two, novel dual-task Stroop variants: Color-Dual (maintain secondary count of prespecified font color regardless the lexical content) and Lexical-Dual (maintain secondary count of prespecified word regardless the font color). With regard to Baseline performance, we predicted an Age x Trial Type interaction in which older adults would be selectively impaired on Incongruent trials compared to younger adults, and this prediction was supported. When we added secondary task demands, we predicted a Trial Type x Dual-Task Type interaction in which performance in the Lexical-Dual condition would be worse than performance in the Color-Dual condition. This prediction was also supported, suggesting that having a secondary task that activated the irrelevant stream of information required more inhibitory control. Finally, we also predicted that Age would interact with Trial Type and Dual-Task Type, which was partially supported in response latencies and more definitively supported in error rates. Overall, our results indicate that Stroop performance is differentially influenced by additional dual-task demands that potentially minimize strategy usage, which has implications for both young and older adult Stroop performance.

## Introduction

On the way to the grocery store, you are stopped at a red light in the left turning lane. The light turns green, and vehicles to your side begin moving forward; however, you realize that you have an additional left turning arrow that is still red. Your ability to overcome the prepotent response to start driving and to inhibit the irrelevant information of cars around you moving forward becomes crucial for your safety on the road. To succeed in this driving example requires a type of executive control called inhibition [1, 2], which is an ability that can differ between age groups [3] and has consequences in both lab and real-world settings. Important for inhibition is the capacity to resist interference and actively maintain goals [4]. Understanding how inhibition and goal maintenance differ across age-groups can help us to better understand and serve adults of any age in applied domains such as transportation or telemedicine.

Inhibition (and executive control more broadly) can be studied with a variety of lab-based measures, and one of the gold standard measures is the Stroop task [5]. There are many versions of the Stroop task, but most involve presenting words on a screen in different font colors along with instructions to respond to the color of the font rather than the lexical content of the word. On congruent trials in which the font color and lexical content match (e.g., BLUE), responses are faster and errors are rarer compared to incongruent trials in which the font color and lexical content mismatch (e.g., BLUE). The average difference in performance across congruent and incongruent trials is commonly referred to as the Stroop effect [6].

Researchers have put forth several theoretical explanations for the Stroop effect [7]. One popular explanation is that our tendency to read visually-presented words is incredibly strong, whereas our tendency to name colors is less automatic; thus, we must inhibit our word reading tendency and instead name the font color, which is particularly difficult in incongruent trials [6, 8]. Despite decades of research, it is not entirely clear whether the locus of the Stroop effect resides at the task set level, semantic level, or response selection level [9]. In addition, some have found evidence of separable neural processes for detecting conflict and resolving conflict in Stroop tasks [10]. Furthermore, from decades of research on the Stroop task, we have learned that performance can vary due to contextual factors, such as circadian rhythm [11] or practice [12] as well as individual differences factors, such as age [13].

Older adults have demonstrated an increased Stroop effect in a number of studies compared to young adults [14–17]. Some have attributed this difference to age-related changes in processing speed [18], yet others have argued it reflects age-related deficits in inhibition [19] that exist even after controlling for processing speed [20] and may be related to age-related changes in frontal neural circuitry [21] and neurobiological functioning [22].

As researchers who regularly use the Stroop task in our labs, we readily admit that it is difficult to successfully respond to the font color rather than the lexical content of stimuli on incongruent trials, and we often hear similar reports from our young and older adult participants. But we have also had young and old participants report that the Stroop task is easy, and when asked to elaborate, they have reported various task strategies, such as using peripheral vision, only focusing on the final letter, or blurring their vision, which undoubtedly helps to minimize processing the lexical content (and thus reduces the need to inhibit this information). In other words, it is possible to game the Stroop task. This presents challenges for construct validity, especially when exploring possible age-related differences in inhibition.

The idea that participants may use strategies to bypass or reduce interference in the Stroop task is not new [23]. Indeed, others have systematically demonstrated that it is possible to reduce the Stroop effect by averting the eyes from centrally presented stimuli or by blurring vision [24]. It can be difficult to monitor and control for these types of strategies, but that does not mean we should not try. Here, we sought to leverage multitasking via a novel dual-task Stroop paradigm to explore age-related differences in inhibitory control in a way that potentially minimized purported strategies participants might use to reduce or bypass Stroop interference.

Our novel Stroop paradigm starts with a traditional single-task Stroop task (Baseline), followed by two dual-task Stroop variants: Color-Dual and Lexical-Dual. In the Color-Dual condition, participants are instructed to complete a traditional Stroop task while also counting the number of times they encounter a stimulus of a certain color, regardless of the lexical content. In other words, the Color-Dual condition adds a secondary task goal that is compatible with the goal of the single-task Stroop task (i.e., attend to font color rather than lexical content). In the Lexical-Dual condition, participants are instructed to complete a traditional Stroop task while also counting the number of times they encounter a prespecified word, regardless the font color. In other words, the Lexical-Dual condition adds a secondary task goal that is incompatible with the single-task Stroop instructions but strongly aligns with our prepotent bias to read lexical content.

We have several predictions based on this novel Stroop paradigm. Starting with the Baseline condition, we expect to find a main effect of Trial Type with performance on incongruent trials being worse than performance on congruent and neutral trials. We also expect to find a main effect of Age with younger adults outperforming older adults. Finally, based on purported age-related differences in inhibitory control, we expect to find an interaction between Age and Trial type such that older adult performance would be selectively worse than young adult performance on incongruent trials.

When we add secondary task demands to the protocol, we still expect to find a main effect of Trial Type similar to Baseline. In addition, we expect to find a main effect of Dual-Task Type with performance on the Color-Dual condition to be better than performance on the Lexical-Dual condition since the Color-Dual condition adds a secondary task goal that is compatible with the single-task Stroop instructions whereas the Lexical-Dual condition adds a secondary task goal that is incompatible with the single-task Stroop instructions. Importantly, we also expect to find an interaction between Trial Type and Dual-Task Type with performance differences across the Color-Dual and Lexical-Dual conditions to be selectively heightened on incongruent trials. Finally, when we consider Age, it is not clear whether older adults would be more impacted (compared to younger adults) across the Color-Dual versus Lexical-Dual conditions. We investigate this by testing for an interaction between Age, Trial Type, and Dual-Task Type. Focusing on key incongruent trials, if we observe more exaggerated effects for older adults compared to younger adults on the Lexical-Dual condition but not the Color-Dual condition, this could suggest that older adults struggle more with inhibitory control in more complex, dual-task settings.

## Methods

### Participants

Thirty-four young adults [range = 18–24 years; mean age = 21 years (sd = 2 years); mean education = 13 years (sd = 2 years); 15 female] and thirty-three older adults [range = 60–80 years; mean age = 66 years (sd = 5 years); mean education = 16 years (sd = 3 years); 16 female] were recruited from the local community to take part in this study. Our sample size was based on prior research involving dual-task Stroop manipulations (albeit different from our novel dual-task Stroop paradigm) with younger adults, and we aimed to have double their sample sizes (i.e., 34 total per age group) since we were interested in age-related effects and a potential 3-way interaction [25, 26]. Participants had normal or corrected-to-normal vision and hearing and were free from neurological disorders or head trauma. We also screened older participants to rule out dementia using the Mini-Mental State Examination [27]. Specifically, older adult participants had to have a score of 27 or higher on the MMSE to be eligible for this study (mean = 28.9; sd = 1.1) [28]. The University of Illinois, Urbana-Champaign Institutional Review Board approved all procedures used in this study (UIUC IRB02065). All participants provided written informed consent and were compensated $8/hr.

### Dual-task Stroop paradigm

**Fig 1:**
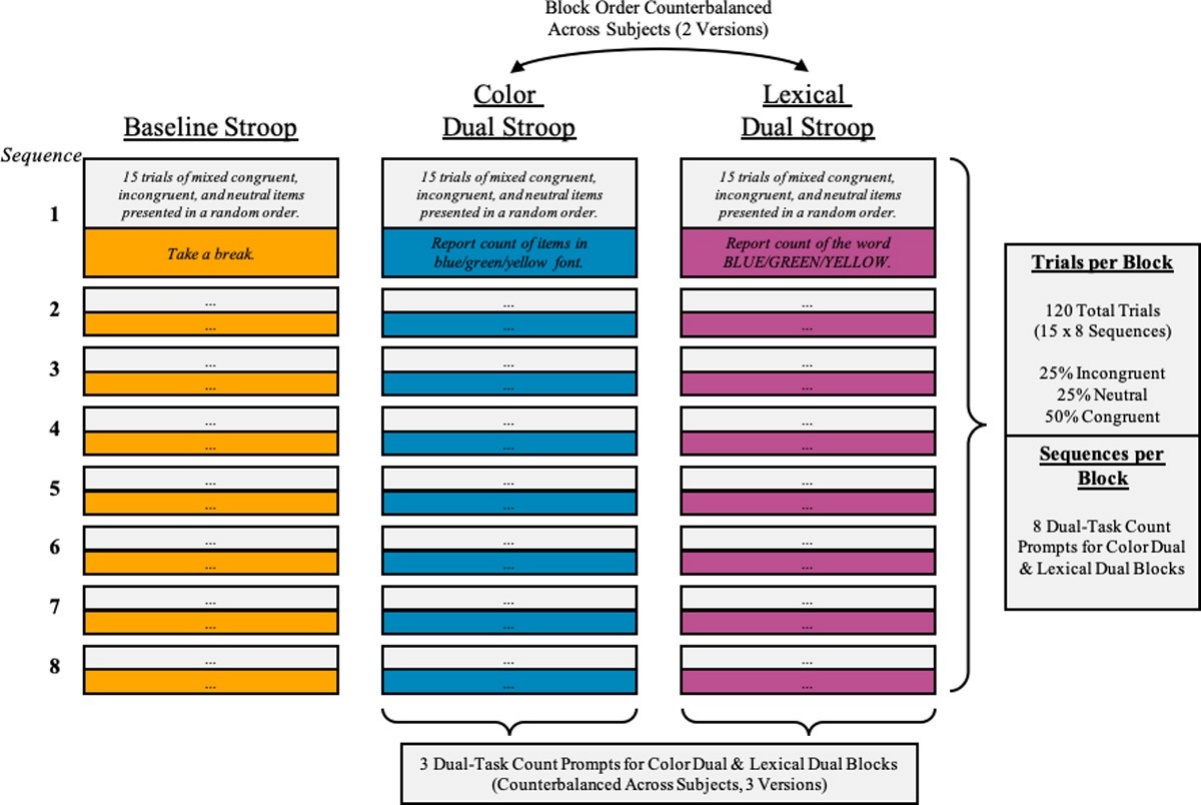
Experimental design schematic.

At the heart of our approach is a modified Stroop task broken into blocks of trials. The trials of all blocks followed the same structure: First, a central fixation for 750ms, then a central stimulus for up to 5000ms to which participants were instructed to indicate the stimulus font color using corresponding color patches over the left, down, and right keys on a keyboard, and finally an interstimulus interval (ISI) of 500ms. Stimuli were randomized color words (BLUE, GREEN, YELLOW) and strings of X’s each presented in blue, green, or yellow font color on a black background. These properties combined to create three different trial types: incongruent (e.g., BLUE), congruent (e.g., BLUE), and neutral (e.g., XXXX). Across an entire block, participants saw 25% incongruent, 25% neutral, and 50% congruent trials [29]. Trials were divided into sequences of 15 that were punctuated by prompts. Each Stroop block contained 8 test sequences, which were always preceded by a practice sequence of 15 trials. This resulted in 135 trials per block (15 practice, 120 test) and 405 trials overall per participant (Fig 1).

We first had participants complete a baseline Stroop task, which allowed us to examine any possible age-related Stroop differences without the addition of a secondary task. Next, participants completed two dual-task Stroop variants: Color-Dual and Lexical-Dual (counterbalanced). In the Color-Dual condition, we instructed participants to respond to the stimulus font color. In addition, we asked them to count the number of times they saw any stimulus in a specific font color (blue, green, or yellow; counterbalanced across participants) regardless the stimulus type. At the end of the 15 trials, we prompted participants to enter their count and then to restart their color count in the next block of trials. The Lexical-Dual condition was nearly identical to the Color-Dual condition except rather than instructing participants to keep a count of items in a certain color, we instructed them to count the number of times they saw a prespecified word (“BLUE”, “GREEN”, or “YELLOW”; counterbalanced across participants) regardless the font color. At the end of the 15 trials, we prompted participants to enter their count and then to restart their lexical count in the next block of trials.

### Procedure

After participants provided informed consent, they sat approximately 50 cm from a Dell monitor to complete the three versions of our Stroop task (Baseline, Color-Dual, and Lexical-Dual). Each version included practice trials during which participants demonstrated proficiency with the response mappings and overall task goals, and we instructed participants to ask questions if they were confused at any point. At the end of the study, participants reflected on the experiment and were offered a debriefing form.

## Results

### Baseline performance

We analyzed baseline response times and error rates using mixed model ANOVAs (jamovi, version 1.2) with a 3 x 2 design which included Trial Type (Congruent vs Incongruent vs Neutral) as a within-subjects factor and Age (Young vs Old) as a between-subjects factor. When sphericity was violated, we applied Greenhouse-Geisser corrections. For significant main effects and significant interactions, we calculated post hoc tests with standard Tukey corrections. To minimize the influence of outliers, we first screened response times [30]. We eliminated any trials faster than 200ms or slower than 4000ms; then we removed any trials that were 3 standard deviations above or below the mean for each participant (this resulted in removal of 1.6% of the total data, or 2.2 average trials per participant). Finally, we removed incorrect trials and computed the mean of the remaining response times for each task condition (Table 1). Baseline inferential statistics are reported in Table 2.

**Table 1:** Baseline descriptive statistics for response latency and accuracy.

**Mean RTs (ms) on Correct Trials**				
	**Older Adults**		**Younger Adults**	
**Trial Type**	**Mean**	**SD**	**Mean**	**SD**
Congruent	865	198	631	112
Incongruent	1117	267	747	140
Neutral	862	182	638	105
**Error Rates**				
	**Older Adults**		**Younger Adults**	
**Trial Type**	**Mean**	**SD**	**Mean**	**SD**
Congruent	0.00	0.00	0.02	0.02
Incongruent	0.02	0.05	0.05	0.04
Neutral	0.00	0.01	0.03	0.04

Note: Older (N = 33) and Younger (N = 34).

**Table 2:** Baseline inferential statistics for response latency and accuracy.

ANOVA Effect	F Value	P Value	Partial η^2^
**Z-Scored RTs**			
Trial Type	211.80	< 0.01	0.77
Age	0.67	0.42	0.01
Trial Type x Age	16.00	< 0.01	0.20
**Error Rates**			
Trial Type	17.11	< 0.01	0.21
Age	17.20	< 0.01	0.21
Trial Type x Age	0.45	0.64	0.01

Note: Older (N = 33) and Younger (N = 34).

#### Z-scored response times

Prior research has suggested that age-related differences in cognitive performance may be the result of general changes in processing speed rather than differences in cognitive performance between age groups [31]. Thus, to control for age-related declines in general processing speed, we conducted our response time analyses on individual z-scored response latencies [32]. More specifically, we calculated these z-scores using each participant’s mean and standard deviation within task but collapsed across conditions [30] (see Supplemental Materials for baseline results based on uncorrected response latencies).

**Fig 2:**
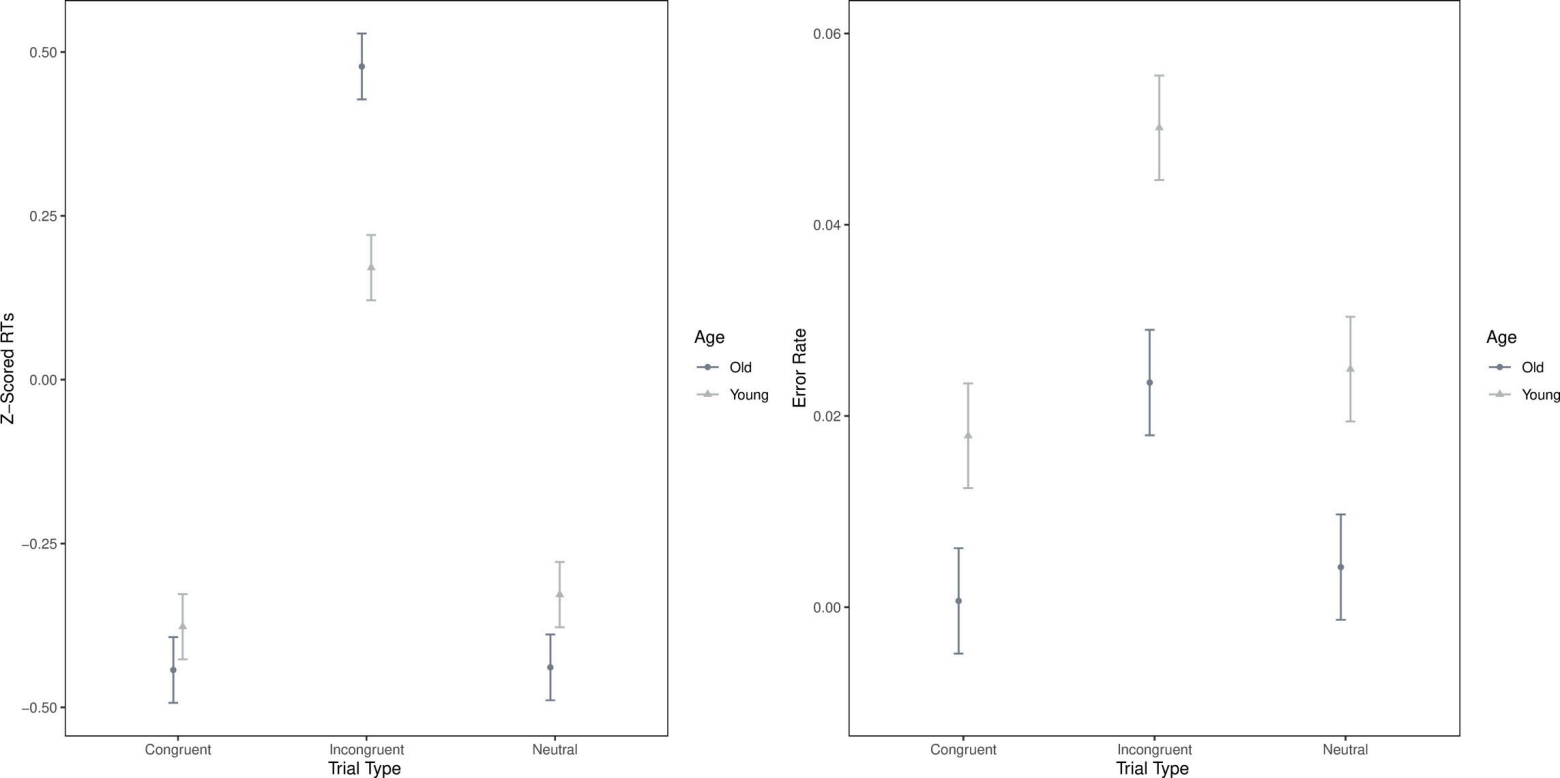
A. Z-scored response times for trial type and age. B. Error rates for trial type and age.

The ANOVA on z-scored RTs yielded several effects (Table 2). We observed a main effect of Trial Type with Incongruent being slower than Congruent and Neutral trials. We did not find a main effect of Age, but there was an interaction between Trial Type and Age such that older adults were significantly more slowed on key Incongruent trials compared to younger adults (t = 4.33; Mean Difference = 0.31; SE = 0.07; p_tukey_ < .001; Fig 2A).

#### Error rates

With regard to error rates, there was a main effect of Trial Type with Congruent and Neutral trials having fewer errors compared to Incongruent trials though they did not differ from each other. There was also a main effect of Age in which older adults were more accurate than younger adults. Whereas the pattern was similar to what we observed with RTs, there was no significant interaction between Trial Type and Age for error rates (Table 2 and Fig 2B).

### Dual-task Stroop performance

#### Primary task analysis

We analyzed dual-task Stroop response times and error rates using mixed model ANOVAs with a 2 x 3 x 2 design which included Dual-Task Type (Color-Dual vs Lexical-Dual) and Trial Type (Congruent vs Incongruent vs Neutral) as within-subjects factors and Age (Young vs Old) as a between-subjects factor. We applied the same filtering as with the baseline response times and error rates, which resulted in removing 1.9% of the total data, or 5.1 average trials per participant (Table 3). Dual-task inferential statistics are reported in Table 4.

**Table 3:** Dual-task descriptive statistics for primary task response latency and accuracy.

**Mean RTs (ms) on Correct Trials**					
		**Older Adults**		**Younger Adults**	
**Dual-Task Type**	**Trial Type**	**Mean**	**SD**	**Mean**	**SD**
Color-Dual	Congruent	934	181	672	145
Color-Dual	Incongruent	1176	299	784	180
Color-Dual	Neutral	939	184	685	144
Lexical-Dual	Congruent	980	181	738	144
Lexical-Dual	Incongruent	1330	268	949	214
Lexical-Dual	Neutral	969	160	729	138
**Error Rates**					
		**Older Adults**		**Younger Adults**	
**Dual-Task Type**	**Trial Type**	**Mean**	**SD**	**Mean**	**SD**
Color-Dual	Congruent	0.01	0.01	0.02	0.02
Color-Dual	Incongruent	0.03	0.05	0.07	0.12
Color-Dual	Neutral	0.00	0.01	0.03	0.03
Lexical-Dual	Congruent	0.00	0.01	0.01	0.02
Lexical-Dual	Incongruent	0.03	0.05	0.13	0.11
Lexical-Dual	Neutral	0.01	0.02	0.02	0.03

Note: Older (N = 33) and Younger (N = 34).

**Table 4:** Dual-task inferential statistics for primary task response latency and accuracy.

ANOVA Effect	F Value	P Value	Partial η^2^
**Z-Scored RTs**			
Dual-Task Type	58.91	< 0.01	0.48
Trial Type	418.75	< 0.01	0.87
Age	0.25	0.62	0.00
Dual-Task Type x Trial Type	57.74	< 0.01	0.47
Dual-Task Type x Age	2.52	0.12	0.04
Trial Type x Age	11.94	< 0.01	0.16
Dual-Task Type x Trial Type x Age	0.78	0.46	0.01
**Error Rates**			
Dual-Task Type	9.80	< 0.01	0.13
Trial Type	28.44	< 0.01	0.30
Age	21.60	< 0.01	0.25
Dual-Task Type x Trial Type	12.07	< 0.01	0.16
Dual-Task Type x Age	8.13	< 0.01	0.11
Trial Type x Age	6.41	< 0.01	0.09
Dual-Task Type x Trial Type x Age	13.73	< 0.01	0.17

Note: Older (N = 33) and Younger (N = 34).

#### Z-scored response times

**Fig 3:**
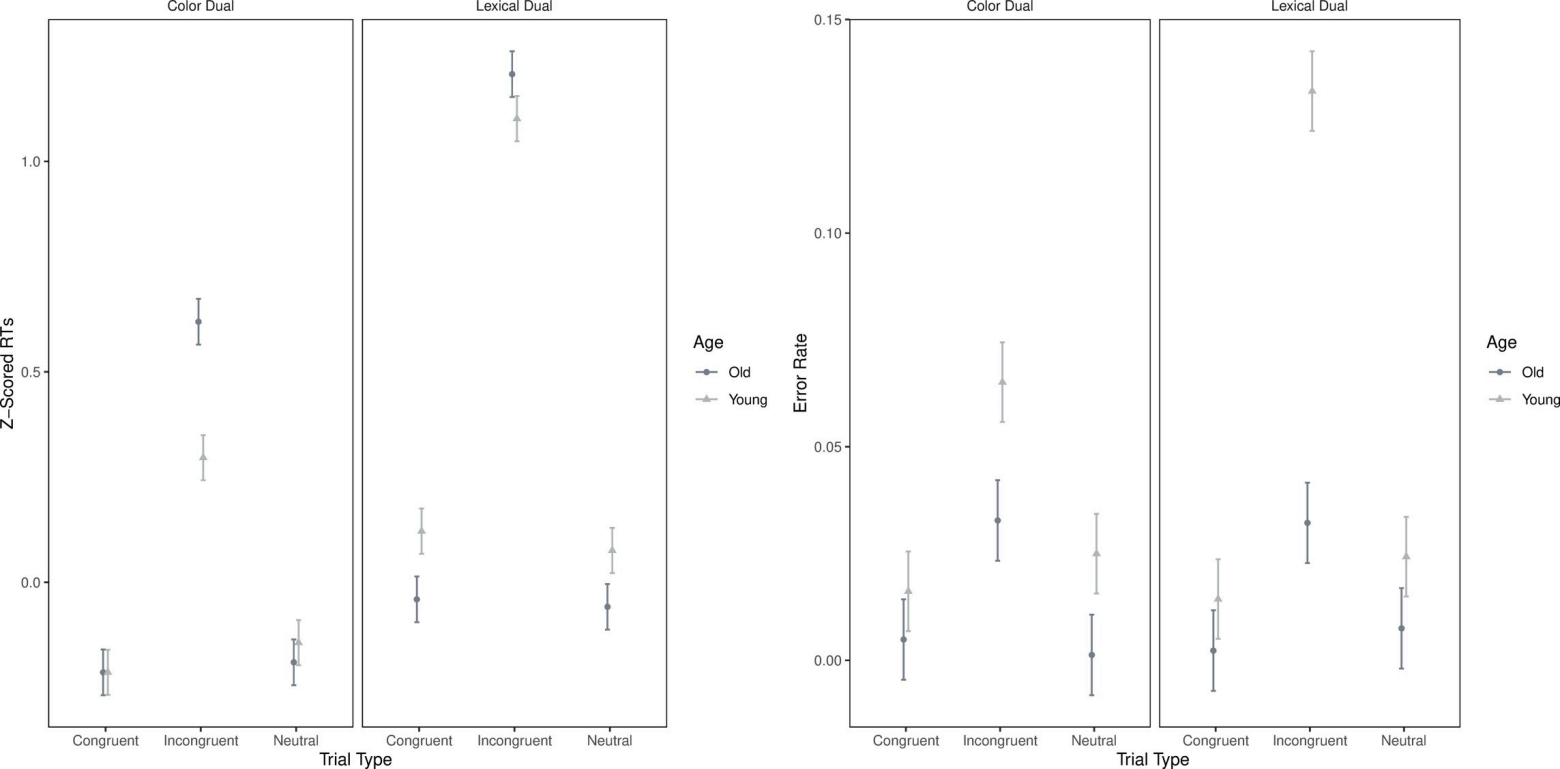
A. Z-scored response times for dual-task type, trial type, and age. B. Error rates for dual-task type, trial type, and age.

Similar to our Baseline Performance analysis, we corrected for any general effects of age-related slowing by z-scoring response times [20, 32] (see Supplemental Materials for dual-task results based on uncorrected response latencies). The ANOVA on z-scored RTs yielded several effects (Table 4). First, there was a main effect of Trial Type with Incongruent being significantly slower than Congruent or Neutral trials. Second, there was a main effect of Dual-Task Type with the Color-Dual condition being significantly faster than the Lexical-Dual condition. Finally, there was an interaction between Dual-Task Type and Trial Type with Incongruent trials in the Lexical-Dual condition being significantly slower than Incongruent trials in the Color-Dual condition (t = -12.12; Mean Difference = -0.70; SE = 0.06; p_tukey_ < .001; Fig 3A).

With regard to Age, we did not observe a main effect; however, there was an interaction between Age and Trial Type with older adults being significantly slower on Incongruent trials compared to younger adults (t = 4.28; Mean Difference = 0.21; SE = 0.05; p_tukey_ < .001). Finally, the 3-way interaction between Age, Trial Type, and Dual-Task Type for response times did not reach significance. However, when we examined the key Incongruent trials, we found that older adults were slower than younger adults in the Color-Dual condition (t = 4.24; Mean Difference = 0.32; SE = 0.08; p_tukey_ = .002), and this difference was not significant in the Lexical-Dual condition (t = 1.39; Mean Difference = 0.11; SE = 0.08; p_tukey_ = .965; Fig 3A).

#### Error rates

With regard to error rates, there was a main effect of Dual-Task Type with the Color-Dual condition being more accurate than the Lexical-Dual condition. There was also a main effect of Trial Type with Incongruent trials being less accurate than Congruent and Neutral trials. Finally, there was an interaction between Dual-Task Type and Trial Type in which error rates were higher on Incongruent trials in the Lexical-Dual condition compared to in the Color-Dual condition (t = -5.76; Mean Difference = -0.03; SE = 0.01; p_tukey_ < .001).

There was a main effect of Age (Older more accurate than Younger), as well as 2-way interactions between Age and Dual-Task Type, and Age and Trial Type, and a 3-way interaction between Age, Dual-Task Type, and Trial Type (Table 4). Interestingly, on key Incongruent trials, older adults were more accurate than younger adults in the Lexical-Dual condition (t = -7.62; Mean Difference = -0.10; SE = 0.01; p_tukey_ < .001) but this difference was not significant in the Color-Dual condition (t = -2.44; Mean Difference = -0.03; SE = 0.01; p_tukey_ = .381; Fig 3B).

#### Secondary task analysis

In addition to analyzing primary task effects, we evaluated whether there were differences in secondary task performance as a function of Age (Young vs Old) and Dual-Task Type (Color-Dual vs Lexical-Dual). Using mixed model analyses of variance (ANOVAs), we ran a 2x2 model on two metrics of secondary task accuracy: Absolute Error Rate (average number of errors on Color-Dual or Lexical-Dual count) and Relative Errors (average magnitude of error on Color-Dual or Lexical-Dual count; e.g., if the correct count was 4 and a participant responded with 5, their relative error would be 1 because 5 is 1 number off from the correct response). Due to a technical error, we only have secondary task data for 32 of the original 33 older adults (Table 5).

**Table 5:** Dual-task descriptive statistics for secondary task counts.

**Absolute Error Rates**				
	**Older Adults**		**Younger Adults**	
**Dual-Task Type**	**Mean**	**SD**	**Mean**	**SD**
Color-Dual	0.66	0.42	0.70	0.38
Lexical-Dual	0.50	0.35	0.63	0.29
**Relative Errors**				
	**Older Adults**		**Younger Adults**	
**Dual-Task Type**	**Mean**	**SD**	**Mean**	**SD**
Color-Dual	0.93	0.60	1.02	0.56
Lexical-Dual	0.66	0.48	0.89	0.45

Note: Older (N = 32) and Younger (N = 34).

#### Count accuracy

The ANOVA of Absolute Error Rate yielded a significant main effect of Dual-Task Type with the Color-Dual condition having a significantly higher error rate than the Lexical-Dual condition; however, there was no significant main effect of Age nor a significant interaction of Age and Dual-Task Type. Similarly, the ANOVA of Relative Errors yielded a significant main effect of Dual-Task Type with the Color-Dual condition having a significantly more relative errors than the Lexical-Dual condition; however, there was still no main effect of Age nor an interaction of Age and Dual-Task Type (Table 6).

**Table 6:** Dual-task inferential statistics for secondary task counts.

ANOVA Effect	F Value	P Value	Partial η^2^
**Absolute Error Rates**			
Dual-Task Type	21.93	< 0.01	0.26
Age	0.95	0.33	0.02
Dual-Task Type x Age	2.99	0.09	0.05
**Relative Errors**			
Dual-Task Type	17.61	< 0.01	0.22
Age	1.73	0.19	0.03
Dual-Task Type x Age	2.00	0.16	0.03

Note: Older (N = 32) and Younger (N = 34).

## Discussion

In this study, we used a novel dual-task Stroop paradigm to explore age-related differences in inhibitory control in a way that potentially minimized purported strategies participants might use to reduce or bypass Stroop interference. A group of young and older adults first completed a baseline Stroop task. Using z-scored response times to control for possible age-related slowing effects unrelated to inhibition [32], we found typical Stroop effects (i.e., main effect of Trial Type) and no main effect of Age. We also found an interaction between Age and Trial Type with older adults seeming to struggle on Incongruent (but not Congruent or Neutral) trials more so than younger adults, which aligned with our original predictions. This also aligns with other work that has found age-related effects in inhibitory control processes using single-task Stroop tasks even after controlling for general age-related slowing effects [20, 33] and perhaps indicates an age-related deficit in inhibitory control [19].

In terms of baseline error rates, we found a main effect of Trial Type, but we did not find an interaction between Trial Type and Age, which was counter to our predictions. We also observed a main effect of Age. Interestingly, older adults had lower error rates than younger adults and thus could be said to outperform younger adults on this metric, which is uncommon but not without precedent on at least some related measures of inhibition [34, 35]. Some have argued that increased response times on incongruent trials reflect inhibitory control processes required for successfully completing the task [36], whereas increased errors reflect the ability to maintain the task goal of naming the font color [4, 37]. Based on our baseline results, it is possible that younger adults perhaps prioritized inhibitory control processes at the expense of goal maintenance whereas older adults had a more balanced allocation of processing resources, although more research is needed for this to be definitive.

For the primary focus of our study, participants completed two novel dual-task Stroop paradigms designed with either a secondary task goal that is compatible with the Stroop task (Color-Dual) or a secondary task goal that is incompatible with the Stroop task (Lexical-Dual). We did this by instructing participants to keep track of any stimuli that were in a certain font color regardless the lexical content (Color-Dual) or any stimuli that were a certain word regardless the font color (Lexical-Dual) and then report those counts at the end of a block of trials. Given that the Lexical-Dual condition activates the irrelevant stream of information participants are supposedly trying to inhibit when completing the Stroop task, we anticipated performance on the Lexical-Dual condition to be worse than performance on the Color-Dual condition selective to Incongruent trials, which is what we found both in terms of response times and error rates.

With regard to response times, we found main effects of Trial Type and Dual-Task Type, as well as an interaction between these factors in which the Lexical-Dual condition was significantly worse than the Color-Dual condition on Incongruent trials. We found similar main effects and interactions on error rates with the Lexical-Dual condition being significantly worse than Color-Dual condition on Incongruent trials. Taken together, it appears as though the type of dual-task matters at a global level since impairments were selective to the Lexical-Dual condition and on Incongruent trials.

When we bring Age into the analysis, our pattern of results becomes somewhat more nuanced. With regard to response times, there was no main effect of Age; however, we did observe an interaction between Age and Trial Type with older adults having slower response times on Incongruent (vs Congruent or Neutral) trials compared to young adults, which was similar to our baseline results. Interestingly, older adults were significantly slower than younger adults on Incongruent trials in the Color-Dual condition, but this difference was not significant in the Lexical-Dual condition. One caveat is that the overall 3-way interaction between Age, Trial Type, and Dual-Task Type for response times did not reach significance. In terms of error rates, Age interacted with Dual-Task Type and Trial Type with older adults having significantly lower error rates on Incongruent trials in the Lexical-Dual condition compared to younger adults, and this difference was not significant in the Color-Dual condition. It is possible that older adults were prioritizing accuracy over response times or were adopting a more cautious response style compared to younger adults [38–40]. On the other hand, it is possible that the younger adults adopted a related strategy in which response times were prioritized over accuracy, which would mirror our baseline single-task Stroop findings at least in terms of error rates. That said, others have pointed out that error rates may result from processes other than inhibition and are thus noisier than response times [41], so this is something that future studies should consider investigating directly. Regardless, the pattern we found for response times in the Color-Dual condition aligns with prior research that has found age-related deficits in dual-task performance, which has led others to theorize that older adults are less able to maintain and coordinate information under conditions requiring divided attention [42, 43]. Interestingly, in the Lexical-Dual condition, Age effects were reduced with both older and younger adults showing comparable slowing on Incongruent trials. It is possible that minimizing the likelihood of using strategies to bypass the Stroop effect (which we argue our Lexical-Dual condition might do) neutralized the difference between older and younger adult performance, although future research is needed to test this directly. It is also possible that by normalizing response times via z-scores, we flattened age-related differences in response time, although this would not have impacted error rates. Thus, we have included results based on uncorrected response latencies in our Supplemental Materials and encourage other researchers to report both uncorrected and corrected RTs in experiments investigating purported age-related differences in inhibitory control [30, 32].

In terms of dual-task Stroop performance on the secondary count task, we found no effects of Age, nor did Age interact with Dual-Task Type, which suggests that both the Color-Dual and Lexical-Dual secondary counting tasks were equally attended to by both young and older adults. Regardless of age group, there was a main effect of Dual-Task Type in which absolute and relative accuracy were lower on the Color-Dual compared to the Lexical-Dual condition. This difference in secondary task errors across the two Stroop tasks may be due “conflict spillage” from the primary task to the secondary counting task. Furthermore, it is possible that the overwhelming lexical bias that generates the Stroop effect may increase when multiple task goals conflict with it (e.g., in the Color-Dual Stroop, *both* the primary and secondary tasks conflict with the lexical bias; whereas in the Lexical-Dual Stroop, only the primary task conflicts with the lexical bias). Whatever the explanation, having participants keep track of information across trials or maintain running counts is perhaps too broad of an approach, though not without precedent [44, 45]. Future studies should consider using a trial-by-trial method of tracking accuracy, perhaps similar to other divided attention tasks like complex span tasks [46].

Inhibition is an important cognitive ability, and the Stroop task is supposedly one way of measuring it. Here we found some age-related differences in performance that may result from underlying differences in inhibitory control. But not all studies using Stroop and different age groups have found age-related differences, especially after accounting for general age-related slowing [47]. For example, in a meta-analysis on age-related Stroop performance, age effects were related to developmental changes in processing speed rather than changes in inhibitory abilities [48], and this aligned with previous structural equation modeling efforts [49]. There have been more recent meta-analyses that have found little to no age-related performance differences on Stroop, though one did find age-related differences on a select group of other inhibition measures [47], as well as age-related differences in more complex experimental conditions, such as dual-tasking [42]. Despite the debate about age-related differences in Stroop performance and whether or not differences that are observed can be attributed to differences in inhibitory control, performance on the Stroop task has continually been used in many labs to index inhibition and in clinical settings as an indicator of cognitive decline [50]. This is somewhat surprising considering there is still debate on the locus of the Stroop effect. For example, in the present study, we are unable to dissociate whether our effects are due to resolving conflict versus detecting conflict in Stroop tasks, which have been shown to rely on separable mechanisms [10, 19, 51]. Thus, future studies should consider using trial-by-trial cueing designs paired with cognitive neuroscientific methods (e.g., EEG, eye tracking) to better tease apart any possible age-related effects in detection versus resolution of conflict in Stroop tasks [52].

We acknowledge that there are certainly other reasons for the discrepancies across studies focusing on age-related differences in Stroop performance, such as differences in the congruency ratios, the type of neutral condition used, the response modality, and many other factors [4, 6, 53]. Here we found yet another possible piece of the puzzle in which two dual-task Stroop paradigms differed in age-related inhibitory control effects. These dual-task Stroop variants were inspired by prior work on strategies participants might use to try to make the Stroop task easier [23, 24]. Indeed, when we asked participants at the end of the study about any strategies they might have used on the task, three older adults and seven younger adults reported using some type of strategy that involved blurring vision, using peripheral vision, looking past the words, though we do not have the precision to know on which conditions they found these strategies more or less successful. Future studies should consider more direct, real-time metrics for tracking strategy implementation to further explore their impact on age-related differences in Stroop performance. This is especially so, since to date our study is the first to use a dual-task approach with secondary task goals that are either compatible or incompatible with the primary Stroop task goals to better understand purported age-related differences in Stroop performance.

## Supporting information

S1 File PLOS ONE data. (CSV)

S2 File Supplemental materials. (PDF)
