# Supplementary material for: Age-related effects on a novel dual-task Stroop paradigm
Source: PLoS One. 2021 Mar 2;16(3):e0247923. doi: 10.1371/journal.pone.0247923 (PMC7924780; doi:10.1371/journal.pone.0247923)
Supplement: S2 File — (PDF) [file pone.0247923.s002.pdf]

Baseline: Uncorrected Response Times

The ANOVA on uncorrected RTs yielded several effects (Table 1). First, there was a main effect of Trial Type in which both Congruent and Neutral trials were faster than Incongruent trials but did not significantly differ from each other. Second, there was a main effect of Age and an interaction between Trial Type and Age with older adults exhibiting significantly more slowing on Incongruent trials compared to younger adults.

Table 1. Baseline inferential statistics for response latency (uncorrected)

ANOVA Effect	F Value	P Value	Partial η^2
**Mean RTs**			
Trial Type	161.90	< 0.01	0.71
Age	45.80	< 0.01	0.41
Trial Type x Age	24.30	< 0.01	0.27

Note: Older (N=33) and Younger (N=34).

Dual-Task: Uncorrected Response Times

The ANOVA on uncorrected RTs yielded several effects (Table 2). First, there was a main effect of Dual-Task Type in which responses were faster on the Color-Dual condition compared to the Lexical-Dual condition. Second, there was a main effect of Trial Type and an interaction between Dual-Task Type and Trial Type such that Incongruent trials were more slowed for the Lexical-Dual condition compared to the Color-Dual condition. There was a main effect of Age, as well as an interaction between Age and Trial Type with Incongruent trials being particularly slowed for older adults compared to younger adults.

Table 2. Dual-task inferential statistics for primary task response latency (uncorrected)

ANOVA Effect	F Value	P Value	Partial η^2
**Mean RTs**			
Dual-Task Type	45.83	< 0.01	0.41
Trial Type	224.94	< 0.01	0.78
Age	48.80	< 0.01	0.43
Dual-Task Type x Trial Type	48.29	< 0.01	0.43
Dual-Task Type x Age	0.34	0.56	0.01
Trial Type x Age	20.36	< 0.01	0.24
Dual-Task Type x Trial Type x Age	0.06	0.94	0.00

Note: Older (N=33) and Younger (N=34).

Additional 3-Level Analyses

The following tables contain the results of 3 x 3 x 2 ANOVAs with Task Type (Baseline vs Color-Dual vs Lexical-Dual) and Trial Type (Incongruent vs Congruent vs Neutral) as within-subjects factors and Age group as a categorical between-subjects factor. Please note that the Baseline Stroop always came before either Dual-Task Stroop, so these results should be interpreted with caution.

Table 3. Inferential statistics for primary task response latency (uncorrected)

ANOVA Effect	F Value	P Value	Partial η^2
**Mean RTs (Uncorrected)**			
Task Type (Baseline; Color-Dual; Lexical-Dual)	59.48	< 0.01	0.48
Trial Type	251.65	< 0.01	0.80
Age	51.20	< 0.01	0.44
Task Type x Trial Type	30.14	< 0.01	0.32
Task Type x Age	0.54	0.59	0.01
Trial Type x Age	26.67	< 0.01	0.29
Task Type x Trial Type x Age	0.05	0.99	0.00

Note: Older (N=33) and Younger (N=34).

Table 4. Inferential statistics for primary task response latency (corrected)

ANOVA Effect	F Value	P Value	Partial η^2
**Mean RTs (Corrected)**			
Task Type (Baseline; Color-Dual; Lexical-Dual)	71.68	< 0.01	0.52
Trial Type	482.77	< 0.01	0.88
Age	6.84	0.01	0.10
Task Type x Trial Type	34.61	< 0.01	0.35
Task Type x Age	1.40	0.25	0.02
Trial Type x Age	19.19	< 0.01	0.23
Task Type x Trial Type x Age	0.82	0.51	0.01

Note: Older (N=33) and Younger (N=34).

Table 5. Inferential statistics for primary task error rates

ANOVA Effect	F Value	P Value	Partial η^2
**Error Rates**			
Task Type (Baseline; Color-Dual; Lexical-Dual)	7.83	< 0.01	0.12
Trial Type	35.12	< 0.01	0.35
Age	28.60	< 0.01	0.31
Task Type x Trial Type	9.30	< 0.01	0.13
Task Type x Age	4.67	0.01	0.07
Trial Type x Age	6.05	< 0.01	0.09
Task Type x Trial Type x Age	7.92	< 0.01	0.12

Note: Older (N=33) and Younger (N=34).
